# New Books

**Published:** 2005-11

**Comprehensive Toxicology: General Principles**

J.A. Bond

Burlington, MA:Elsevier, 2005. 354 pp. ISBN: 0-08-042966-1, $146.95

**Comprehensive Toxicology: Renal Toxicology**

R.S. Goldstein

Burlington, MA:Elsevier, 2005. 716 pp. ISBN: 0-08-042972-6, $196.95

**Deliberative Environmental Politics: Democracy and Ecological Rationality**

Walter F. Baber, Robert V. Bartlett

Cambridge, MA:MIT Press, 2005. 288 pp. ISBN: 0-262-02587-6, $60

**Environmental Chemistry: Chemistry of Major Environmental Cycles**

Teh Fu Yen

Hackensack, NJ:World Scientific Publishing, 2005. 640 pp. ISBN: 1-86094-474-4, $76

**Environmental Citizenship**

Andrew Dobson, Derek Bell

Cambridge, MA:MIT Press, 2005. 312 pp. ISBN: 0-262-02590-6, $60

**Global Change and the Earth System: A Planet Under Pressure**

W. Steffen, S. Sanderson, P.D. Tyson, J. Jäger, P.A. Matson, B. Moore III, et al.

New York:Springer-Verlag, 2005. 332 pp. ISBN: 3-540-26594-5, $139

**Governing Water: Contentious Transnational Politics and Global Institution Building**

Ken Conca

Cambridge, MA:MIT Press, 2005. 456 pp. ISBN: 0-262-03339-9, $70

**Hot Spot Pollutants: Pharmaceuticals in the Environment**

Daniel Dietrich, Simon Webb, Thomas Petry

Burlington, MA:Elsevier, 2005. 352 pp. ISBN: 0-12-032953-0, $190

**Industrial Transformation: Environmental Policy Innovation in the United States and Europe**

Theo de Bruijn, Vicki Norberg-Bohm, eds.

Cambridge, MA:MIT Press, 2005. 376 pp. ISBN: 0-262-54181-5, $27

**Inventing for the Environment**

Arthur Molella, Joyce Bedi, eds.

Cambridge, MA:MIT Press, 2005. 424 pp. ISBN: 0-262-63328-0, $17.95

**Seeing the Forest and the Trees: Human-Environment Interactions in Forest Ecosystems**

Emilio F. Moran, Elinor Ostrom, eds.

Cambridge, MA:MIT Press, 2005. 504 pp. ISBN: 0-262-13453-5, $83

**Small-Scale Freshwater Toxicity Investigations: Vol. 1—Toxicity Test Methods**

Christian Blaise, Jean-François Férard, eds.

New York:Springer-Verlag, 2005. 551 pp. ISBN: 1-4020-3119-X, $129

**Small-Scale Freshwater Toxicity Investigations: Vol. 2—Hazard Assessment Schemes**

Christian Blaise, Jean-François Férard, eds.

New York:Springer-Verlag, 2005. 551 pp. ISBN: 1-4020-3543-8, $129

**Sustainability and Cities: Concept and Assessment**

Ooi Giok Ling

Hackensack, NJ:World Scientific Publishing, 2005. 252 pp. ISBN: 981-256-163-3, $48

**The Law and Ethics of the Pharmaceutical Industry**

M.N.G. Dukes

Burlington, MA:Elsevier, 2005. 450 pp. ISBN: 0-444-51868-1, $129.95

**Worldviews, Science and Us: Redemarcating Knowledge and Its Social and Ethical Implications**

Diederik Aerts, Bart D’Hooghe, Nicole Note, eds.

Hackensack, NJ:World Scientific Publishing, 2005. 232 pp. ISBN: 981-256-190-0, $58

